# Lotilaner Ophthalmic Solution, 0.25%, for the Treatment of *Demodex* Blepharitis

**DOI:** 10.3390/healthcare12151487

**Published:** 2024-07-26

**Authors:** Pinakin Gunvant Davey, Marjan Farid, Paul Karpecki, Ian Benjamin Gaddie, Arthur Chan, James Mun, Sesha Neervannan, Elizabeth Yeu

**Affiliations:** 1 College of Optometry, Western University of Health Sciences, Pomona, CA 91766, USA; contact@pinakin-gunvant.com; 2 Gavin Herbert Eye Institute, University of California-Irvine, Irvine, CA 92697, USA; mfarid@hs.uci.edu; 3 Kentucky Eye Institute, Lexington, KY 40517, USA; karpecki@karpecki.com; 4 Gaddie Eye Centers, Louisville, KY 40222, USA; ibgaddie@me.com; 5 Tarsus Pharmaceuticals, Inc., Irvine, CA 92618, USA; arthur@tarsusrx.com (A.C.); jmun@tarsusrx.com (J.M.); sesha@tarsusrx.com (S.N.); 6 Virginia Eye Consultants, Norfolk, VA 23502, USA

**Keywords:** *Demodex*, blepharitis, dry eye, lotilaner

## Abstract

*Demodex* blepharitis, a chronic lid margin disease, is caused by an infestation of *Demodex* mites, the most common ectoparasites in human skin and eyelids. Lotilaner ophthalmic solution, 0.25% (Xdemvy, Tarsus Pharmaceuticals), is the first therapy approved to treat *Demodex* blepharitis. This narrative review characterizes lotilaner ophthalmic solution, 0.25%, and describes its efficacy, safety, and tolerability. The safety and efficacy of lotilaner ophthalmic solution, 0.25%, for treating *Demodex* blepharitis was evaluated in four phase 2 and two phase 3 trials. The data of 980 patients included in these phase 2 and 3 clinical trials revealed that the proportion of eyes with a clinically meaningful reduction to 10 or fewer collarettes (the cylindrical, waxy debris found at the base of the eyelashes) ranged from 81 to 93%. The mite eradication rate confirmed by a microscopy of epilated lashes ranged from 52 to 78%. No serious treatment-related adverse events were reported in any of these clinical studies. As high as 92% of the patients receiving lotilaner eyedrops in the phase 3 trials found it to be neutral to very comfortable. Given the positive safety and efficacy outcomes, the drug is likely to become the standard of care in the treatment of *Demodex* blepharitis.

## 1. Introduction

Lotilaner ophthalmic solution, 0.25% (Xdemvy, Tarsus Pharmaceuticals), is the first treatment approved by the FDA for the treatment of *Demodex* blepharitis, a condition that affects about 58% of all eye care patients, or about 25 million U.S. adults [1,2,3]. *Demodex* blepharitis, a chronic lid margin disease, is caused by an infestation of *Demodex* mites, the most common ectoparasites in human skin and eyelids. When residing in the hair follicles of the eyelids, the mites cause direct damage through mechanical burrowing and laying eggs, irritation from released chemicals, including digestive enzymes and waste products, and bacterial buildup from harboring and acting as vectors for bacteria in the eye [1,4,5]. Clinical manifestations of *Demodex* blepharitis include collarettes, lid margin erythema, lid and lash abnormalities, chalazia, pterygia, and contact lens intolerance [4,5,6,7,8]. The disease has significant clinical, functional, and psychosocial effects on patients, with more than 77% of patients with confirmed *Demodex* blepharitis indicating that the disease has negatively affected their daily lives [9]. It is prevalent in both sexes and across racial groups [10,11,12].

*Demodex* blepharitis is often intertwined with dry eye disease. A poor-quality tear film due to dry eye disease could make conditions more hospitable for *Demodex* mites [13]. Conversely, the overgrowth of *Demodex* mites can complicate dry eye disease and exacerbate symptoms [14]. Patients using prescription eyedrops for dry eyes are just as likely as those not using such medications to have signs of *Demodex* blepharitis, suggesting that dry eye treatments alone do not mitigate *Demodex* blepharitis [12]. Although there is a significant overlap in symptoms between dry eye disease and *Demodex* blepharitis, dry eye treatments may not provide any relief for the patient’s symptomatic burden [9,13].

Recent studies suggest a costly and substantial burden of illness in patients with *Demodex* blepharitis and highlight the unmet needs in diagnosing and managing *Demodex* blepharitis [9,15]. Nearly all patients (99.2%) with confirmed *Demodex* blepharitis have at least one symptom, and a large majority (96.9%) report three or more symptoms [9]. The results of a patient survey of 113 patients with *Demodex* blepharitis in the U.S. demonstrated that patients have often experienced delays in diagnosis, multiple healthcare provider (HCP) visits, unresolved symptoms, and high costs of disease management [16]. Eye redness, dryness, and itchy eyes/eyelids were some of the chief symptoms that prompted patients to visit their HCP prior to their *Demodex* blepharitis diagnosis [16]. Despite using several management options like warm compresses, artificial tears, and tea-tree-oil-based lid hygiene products, most patients reported unresolved *Demodex* blepharitis [16]. Healthcare resource utilization was also reported to be burdensome in this patient survey. Before diagnosis, patients reported an average of 3.5 HCP visits and a mean of 1.2 years between the appearance of symptoms and diagnosis [16]. After diagnosis with *Demodex* blepharitis, patients continued to visit HCPs multiple times a year due to unresolved disease [16]. More than 50% of *Demodex* blepharitis patients reported visiting an urgent care facility in the previous 12 months due to their *Demodex* blepharitis [16]. Prior to the availability of lotilaner ophthalmic solution, 0.25%, patients reported spending USD 192, on average, for doctor visits, USD 115 for over-the-counter products, USD 175 for prescription medications, and USD 368 for in-office procedures to address their *Demodex* blepharitis symptoms. Additionally, previous management strategies have been shown to provide only limited relief [17,18,19,20,21].

This review characterizes the new agent, lotilaner ophthalmic solution, 0.25%, by its chemical structure and describes the efficacy, safety, and tolerability outcomes seen in the formal clinical trial program for the drug.

## 2. Lotilaner: The First-in-Class Treatment for *Demodex* Blepharitis

Isoxazolines are a family of compounds shown to have acaricidal effects against fleas and ticks [22,23,24]. Isoxazolines are potent inhibitors of gamma-aminobutyric acid (GABA)-gated chloride channels, blocking the transport of chloride ions across cell membranes [25]. Ectoparasites exposed to isoxazolines exhibit spastic paralysis, leading to their starvation and death [26].

**Figure 1:**
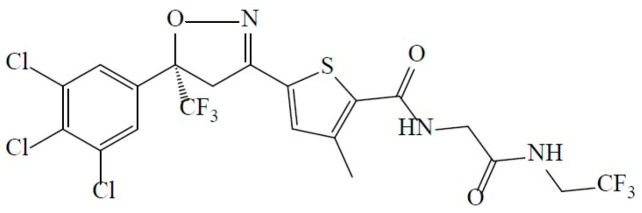
Chemical structure of lotilaner.

Lotilaner, i.e., 2-Thiophenecarboxamide, 5-[(5S)-4,5-dihydro-5-(3,4,5-trichlorophenyl)-5-(trifluoromethyl)-3-isoxazolyl]-3-methyl-N-[2-oxo-2-[(2,2,2-trifluoroethyl)amino]ethyl]-2-thiophenecarboxamide (Figure 1), is a member of the isoxazoline class with a molecular weight of 596.76 g/mol and a measured log Pow (octanol/water partition coefficient) of 5.3 [26]. The lotilaner molecule has a high logP value, making it more soluble in lipophilic, organic solvents than in an aqueous solution. The activity of lotilaner is specific to insect and acari neuroreceptors; its best-in-class lack of effect on the mammalian nervous system at clinically relevant doses has been confirmed in numerous laboratory and target animal safety studies [26]. The lipophilic nature of the lotilaner molecule is thought to promote its preferential uptake into the lid margin and specifically the oily sebum of the eyelash follicles where the *Demodex* mites reside [27].

In well-controlled laboratory and field studies, an oral veterinary formulation of lotilaner was shown to be >98% effective against fleas and ticks in dogs and cats [28,29]. Efficacy in dogs was demonstrated as soon as 2 h after administration, with the ability to maintain efficacy against subsequent re-infestations for at least 35 days after initial administration [30,31]. Likewise, in cats, oral administration of lotilaner resulted in rapid onset of flea- and tick-killing activity with consistent and sustained efficacy for at least one month [32].

Lotilaner has been marketed since 2018 as Credelio chewable tablets (Elanco). Both the U.S. FDA [33], and the European Medicines Agency (EMA) have concluded that isoxazoline veterinary products are safe and effective [34].

## 3. Lotilaner in Humans for the Treatment of *Demodex* Blepharitis

*Demodex* mites are microscopic ectoparasites of the phylum Arthropoda with a semi-transparent, elongated body and four pairs of legs [1,3,35]. The mites consume epithelial cells at the eyelash follicle and induce epithelial hyperplasia and hyperkeratinization, subsequently leading to the formation of collarettes (also known as cylindrical dandruff) [1,5,18]. Collarettes are the pathognomonic sign of *Demodex* blepharitis and can be readily diagnosed by having the subject look down during a routine slit-lamp examination.

An innovative multidose eyedrop formulation of lotilaner (Xdemvy, lotilaner ophthalmic solution, 0.25%, Tarsus Pharmaceuticals) was approved by the U.S. FDA in July 2023 for the treatment of *Demodex* blepharitis, following the successful conclusion of an extensive clinical trial program. Prior to the clinical trials leading to the approval of Xdemvy (formerly known as TP-03), lotilaner had not been used in humans, let alone for any ophthalmic indications.

The recommended human ophthalmic dose is one drop in each eye twice daily for 6 weeks (42 days). Assuming a 35 μL drop size, four drops per day (two per eye) of 0.25% lotilaner would provide a dose of 0.35 mg/day, or a total dose of 0.005 mg/kg/day for a 75 kg person, well below the preclinical NOAEL [36]. In vitro testing demonstrated that lotilaner did not inhibit mammalian GABA-Cl channels at concentrations up to 30 µM (~1100 times the recommended human ophthalmic dose) [36]. The 42-day course of treatment is intended to provide acaricidal dosing across at least two full life cycles of *Demodex* mites. The life cycle of a mite, from egg through larva, nymph, and adult mite, is estimated to be from 14 to 23 days [37,38].

## 4. Clinical Trial Program

Lotilaner ophthalmic solution, 0.25%, for the treatment of *Demodex* blepharitis has been evaluated in four phase 2 trials [39,40,41,42], conducted at Asociación para Evitar la Ceguera en México I.A.P in Mexico City, Mexico, followed by two large, randomized, double-masked, vehicle-controlled U.S. trials, phase 2b/3 Saturn-1 and phase 3 Saturn-2 [43,44]. Two of the early clinical studies evaluated treatment with lotilaner for 28 days [40,41], while all subsequent studies involved a 6-week (42 days) course of twice-daily treatment. In all, 147 patients participated in the phase 2 studies, and another 833 patients participated in the Saturn-1 and Saturn-2 pivotal trials. The design and outcomes of these studies are summarized in Table 1. A pooled analysis of the Saturn-1 and Saturn-2 studies has also been conducted and submitted for publication. In all these studies, subjects were confirmed to have *Demodex* blepharitis prior to enrollment by meeting all of the following criteria in at least one eye: more than 10 collarettes present on the upper eyelid; at least mild lid margin erythema of the upper eyelid, and average mite density of ≥1.5 mites per lash (upper and lower eyelids combined).

The efficacy of lotilaner ophthalmic solution, 0.25%, for the treatment of *Demodex* blepharitis has been evaluated based on the reduction in collarettes at the base of the eyelashes, mite eradication confirmed by microscopy of epilated lashes, and cure of lid margin erythema. Safety was rigorously evaluated in each of the phase 2–3 trials. Additionally, a 1-year follow-up of patients from Saturn-1 has also been reported [45].

Following the publication of data from the clinical trials, four independent systematic reviews and meta-analyses were conducted, with all four concluding that lotilaner ophthalmic solution, 0.25%, is a promising treatment option for patients with *Demodex* blepharitis, with good evidence of safety and efficacy [46,47,48,49].

## 5. Clinical Trial Outcomes

### 5.1. Efficacy

Lotilaner ophthalmic solution, 0.25%, has demonstrated an early onset of action, with highly statistically significant changes compared to the vehicle group as early as day 15 [43,44]. In the Saturn-1 and Saturn-2 pivotal clinical trials, all pre-specified primary and secondary endpoints were met, with statistically significant differences (*p* < 0.0001) between lotilaner ophthalmic solution 0.25% and the vehicle group [43,44].

### 5.2. Reduction in Collarettes

**Figure 2:**
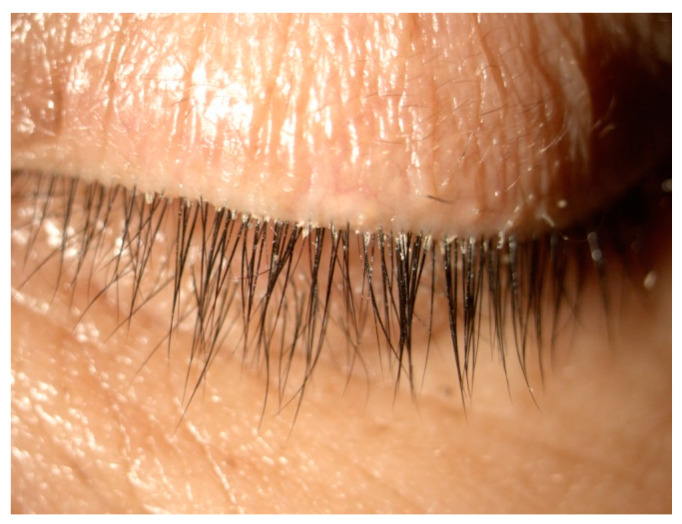
Collarettes are sleeve-like exudative excretions that adhere to the base of the eyelashes.

The primary endpoint in the Saturn-1 and Saturn-2 pivotal clinical trials was reduction in collarettes to 0–2 collarettes. These cylindrical, waxy debris found at the base of the eyelashes (Figure 2) are pathognomonic for *Demodex* blepharitis [4,5,21,50]. Collarettes consist of undigested material, keratinized cells, dead or living mites, and eggs/egg casings [3,39,51]. Because they are easy to visualize under slit-lamp magnification, the presence of collarettes represents the best way to diagnose *Demodex* blepharitis and follow the effects of treatment in routine clinical practice [52].

A grade 0-to-4 collarette scale based on the groupings and/or scales used by Gao et al. and Hosseini et al. [51,53] was first described in a phase 2b study [40] and has been used in all subsequent clinical trials of lotilaner ophthalmic solution, 0.25%. This grading scale is non-linear, with grades 2 to 4 representing much higher levels of *Demodex* infestation (Table 2). A 2-collarette grade improvement—for example, from grade 3 to grade 1—can reflect a 90% reduction in the number of collarettes per eyelid. As shown previously by Gao et al. [51], a reduction in collarettes to ≤10 is associated with reduced mite density and a reduction in the severity of *Demodex* blepharitis. Reducing the number of collarettes to this level, which corresponds to Grade 0–1 on the current grading scale, is considered a clinically meaningful improvement, while a reduction to a collarette grade of 0 (≤2 collarettes) has been considered the most effective measure of success in lotilaner clinical trials.

In the first pivotal study, Saturn-1 [44], lotilaner ophthalmic solution, 0.25%, demonstrated a statistically significantly greater collarette reduction effect compared to the vehicle group. The proportion of patients achieving collarette grade 0 (0–2 collarettes) at day 43 was significantly higher in the lotilaner-treated study group compared to the vehicle control group (44.0% vs. 7.4%, *p* < 0.0001). The proportion of eyes with collarette grade 0–1 (≤10 collarettes) for the upper eyelid of the analysis eye was 81.3% in the study group versus 23.0% in the control group at day 43 (*p* < 0.0001). The mean collarette grade in the study group improved from 2.8 to 0.8 over the course of the study.

Similarly, in the second pivotal study, Saturn-2 [43], the proportion of patients achieving collarette grade 0 (0–2 collarettes) at day 43 was statistically significantly higher in the lotilaner-treated study group compared to the vehicle control group (56.0% vs. 12.5%, *p* < 0.0001). The proportion of eyes with collarette grade 0–1 (≤10 collarettes) for the upper eyelid of the analysis eye was 89.1% in the study group versus 33.0% in the control group at day 43 (*p* < 0.0001). Additionally, 96.4% of lotilaner-treated eyes had at least a 1-grade improvement in collarettes after 6 weeks of treatment.

### 5.3. Mite Eradication

**Figure 3:**
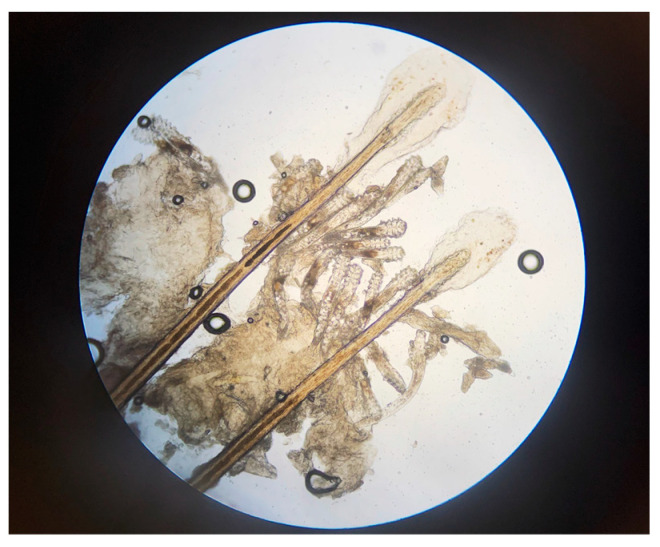
*Demodex* mites shown on epilated eyelashes under magnification (Image courtesy of Dr. Patrick Vollmer, Vita Eye Clinic, Shelby, North Carolina).

*Demodex* mites (Figure 3) can be identified by epilating eyelashes and viewing them under a microscope, although this is impractical in routine clinical practice and not required, as collarettes are a pathognomonic sign of *Demodex* blepharitis [4,5,21,50], as discussed above. In all phase 2–3 studies of lotilaner ophthalmic solution, 0.25%, microscopy confirmation of at least 1.5 mites per lash was required for study inclusion, and mite eradication was an important efficacy measure [40,42,43,44]. In the pivotal Saturn-1 and Saturn-2 trials [43,44], mites were counted at screening and on days 15, 22, and 43 using a slit-lamp biomicroscope to select two or more lashes from the upper and lower eyelids of each eye. Lashes with visible collarettes, if present, were targeted for epilation. The epilated lashes were examined, and mite density was calculated as the number of mites per lash. Eradication was defined as a mite density of 0 mites/eyelash [43,44].

Reductions in mite density occurred early in the course of treatment. By day 43 in the Saturn-1 study, mites had been eradicated in 67.9% of study patients vs. 17.6% of control patients (*p* < 0.0001). Similarly, in the Saturn-2 study, the mite eradication rate was 51.8% in the study group vs. 14.6% in the control group (*p* < 0.0001) at day 43. When complete eradication was not achieved, 94.7% of the study group in Saturn-1 and 86.5% in Saturn-2 had a reduction in mite density to ≤0.5 mites/lash at day 43, compared to 35.8% and 34.7%, respectively, of the control groups in the two studies. The differences between the study and control groups were highly statistically significant [43,44].

### 5.4. Erythema Cure

Erythema of the eyelid margin, caused by chronic inflammation, is a common clinical sign of blepharitis [54,55]. Erythema may also be noted as a symptom by patients, who complain that it negatively affects their physical appearance, potentially influencing social and professional interactions [9].

An erythema grading scale of 0 to 3 (Table 2) was used in the phase 2 Europa clinical trial and all subsequent clinical trials of lotilaner ophthalmic solution, 0.25% [42]. The rates of erythema cure (reduction in redness to grade 0 in the upper eyelid of the analysis eye), as well as a composite grade of 0 for both erythema and collarettes, were evaluated in both pivotal Saturn clinical trials.

In the Saturn-1 trial, the erythema cure rate at day 43 was 19.1% in the study group vs. 6.9% in the vehicle group (*p* = 0.0001). The composite grade 0 rate for both collarettes and erythema was 13.9%, which was significantly higher than in the control group (1.0%, *p* < 0.0001). In Saturn-2, researchers again found statistically significant differences (*p* < 0.0001) between lotilaner ophthalmic solution and vehicle groups in erythema cure (31.1% vs. 9.0%) and a composite grade of 0 (19.2% vs. 4.0%) at day 43.

### 5.5. Safety and Tolerability

#### 5.5.1. Adverse Events

No serious treatment-related adverse events have occurred in any of the human clinical studies of lotilaner ophthalmic solution, 0.25%, to date [39,40,41,42,43,44].

Most ocular treatment-emergent adverse events (TEAEs) in the Saturn pivotal trials were mild, and none were considered serious; the most common was instillation site pain (11.8% in Saturn-1 and 7.9% in Saturn-2) [43,44]. Other ocular adverse reactions worth noting included chalazion/hordeolum (stye) and punctate keratitis, both reported in less than 2% of patients [36]. There were no adverse safety signals on multiple safety measures evaluated in the Saturn-1 and Saturn-2 clinical trials, including intraocular pressure, endothelial cell density, corneal staining, slit-lamp biomicroscopy, fundus examination, or distance visual acuity. Additionally, there were no clinically relevant changes from baseline in median values for systemic clinical laboratory values, including hematology, clinical chemistry, and urinalysis [43,44].

#### 5.5.2. Drop Comfort

Drop comfort on instillation can affect patient compliance and, therefore, the efficacy of treatment. In Saturn-1 and Saturn-2, the drop comfort of lotilaner ophthalmic solution, 0.25%, was assessed at all visits. Patients rated the comfort of the study medications as very comfortable, slightly comfortable, neither comfortable nor uncomfortable, slightly uncomfortable, or very uncomfortable. Most patients (91.9% in Saturn-1 and 90.7% in Saturn-2) in the study group found the drops to be neutral to very comfortable at day 43 [43,44]. There was no significant difference in drop comfort between the study group and vehicle control.

#### 5.5.3. Drug Compliance

Patient compliance with eyedrop administration was verified through an in-office review of patients’ daily diaries at each study visit. Noncompliance or overcompliance with the drug administration schedule was defined as having less than 80% or more than 125% of the expected number of eyedrop administrations [42]. Patients reported high compliance with the drop regimen (mean of 98.6%), which may have been due in part to good tolerability of the drops and their negligible effect on vision.

#### 5.5.4. Long-Term Safety and Duration of Response

The long-term safety and duration of response beyond the recommended 42-day treatment period are of interest to prescribing clinicians. Following the successful completion of the Saturn-1 study, participants who completed that study were invited to participate in an extension study in which they would be monitored for an additional 46 weeks, or 1 year from the initiation of treatment with lotilaner ophthalmic solution, 0.25%, to observe any incidence of long-term safety issues [45]. The duration of response out to 1 year after the 6-week treatment was also observed and evaluated. In all, 239 patients participated in the extension study, during which no additional intervention was administered, and no restriction related to the use of concomitant medications or therapies was enforced.

Only one treatment-related ocular adverse event (blurred vision) occurred in the study group (0.8%) during the 1-year duration of the study, and it was not considered serious. While no ocular serious adverse events (SAEs) were observed in the study group, the two non-ocular SAEs (hip fracture and hematuria) that occurred in the study group were determined to be not related to the study drug. As such, no long-term safety concerns for the study drug were observed in the 1-year extension study [45].

In addition, a statistically significantly (*p* < 0.0001) higher proportion of patients treated with lotilaner ophthalmic solution, 0.25%, for 6 weeks had 0–2 collarettes (grade 0) and ≤10 collarettes (collarette grade of 0 or 1) at every assessment timepoint throughout the 1-year extension study compared to patients who received vehicle control [45].

Furthermore, a continuous improvement in lid margin erythema was observed in the extension study. The proportion of patients in the study group with erythema cure (grade 0) was 21% at day 180 and 29% at day 365, compared to 19% at day 43 in the precursor study. The continuous improvement in erythema after treatment cessation suggested that it may take more time for inflammation to resolve or improve once there is a substantial reduction in mite infestation and collarettes [45].

## 6. Conclusions

To date, lotilaner ophthalmic solution, 0.25%, dosed twice daily as an eye drop, is the only formulation of lotilaner that has been extensively studied in humans. A topical aqueous gel formulation (TP-04, Tarsus Pharmaceuticals) for the potential treatment of papulopustular rosacea is currently being studied in a Phase 2a trial. As the first FDA-approved treatment for *Demodex* blepharitis, lotilaner ophthalmic solution, 0.25%, offers patients an alternative to previous symptom management options that have not been proven safe and effective. Lotilaner ophthalmic solution, 0.25%, was subjected to a robust clinical trial program and demonstrated safety and efficacy, including in the two successful randomized, double-masked, vehicle-controlled pivotal clinical trials enrolling more than 800 patients. Given the positive safety and efficacy outcomes reported in the published literature to date, it is expected that the drug is likely to become the standard of care in the treatment of *Demodex* blepharitis.

## Figures and Tables

**Table 1:** Summary of clinical trial outcomes for lotilaner ophthalmic solution, 0.25%.

Publication	Study	Sample Size (N)	# of Sites	Country	Drug/Dose	Outcome Measures	Safety
Gonzalez-Salinas R et al. (MARS) 2021 [42]	Single-arm, prospective	15	1	Mexico	Lotilaner 0.25% 1 drop OU BID for 28 days	Mite eradication: 57.1%	No AEs
Gonzalez-Salinas R et al. (IO) 2021 [40]	Single-arm, prospective	18	1	Mexico	Lotilaner 0.25% 1 drop OU BID for 42 days	Collarette grade 0: 72.2% Mite eradication: 77.8%	5 drug-related TEAEs: mild blurriness (n = 1); mild burning (n = 4)
Gonzalez-Salinas R et al. (JUPITER) 2022 [41]	Randomized controlled trial (Phase 2b)	60	1	Mexico	Lotilaner 0.25% 1 drop OU BID for 28 days	Collarette grade 0–1: 87.5% (lotilaner) vs. 22.2% (vehicle), *p* < 0.001 Mite eradication Day 28: 66.7% (lotilaner) vs. 25.9% (vehicle), *p* = 0.005	No SAEs; no drug-related AEs
Yeu E et al. (EUROPA) 2023 [43]	Randomized controlled trial (Phase 2b)	54	1	Mexico	Lotilaner 0.25% 1 drop OU BID for 42 days	Collarette grade 0: 80.0% (lotilaner) vs. 15.8% (vehicle), *p* < 0.001 Collarette grade 0–1: 93.3% (lotilaner) vs. 31.6% (vehicle), *p* = 0.0003 Mite eradication day 42: 73.3% (lotilaner) vs. 21.1% (vehicle), *p* = 0.003	No SAEs; 4 drug-related mild AEs: burning (n = 2); burning with red eyes/blurriness (n = 1); change in taste sensation for a few hours (n = 1)
Yeu E et al. (SATURN-1) 2023 [45]	Randomized controlled trial (Phase 2b/3)	421	15	U.S.	Lotilaner 0.25% 1 drop OU BID for 42 days	Collarette grade 0: 44.0% (lotilaner) vs. 7.4% (vehicle), *p* < 0.0001 Collarette grade 0–1: 81.3% (lotilaner) vs. 23.0% (vehicle) *p* < 0.0001 Mite eradication: 67.9% (lotilaner) vs. 17.6% (vehicle), *p* < 0.0001 Erythema cure: 19.1% (lotilaner) vs. 6.9% (vehicle), *p* = 0.0001	No related SAEs; most commonly reported drug-related AE: instillation site pain: 11.8% (lotilaner) vs. 7.7% (vehicle)
Gaddie IB et al. (SATURN-2) 2023 [44]	Randomized controlled trial (Phase 3)	412	21	U.S.	Lotilaner 0.25% 1 drop OU BID for 42 days	Collarette grade 0: 56.0% (lotilaner) vs. 12.5% (vehicle), *p* < 0.0001 Collarette grade 0–1: 89.1% (lotilaner) vs. 33.0% (vehicle), *p* < 0.0001 Mite eradication: 51.8% (lotilaner) vs. 14.6% (vehicle), *p* < 0.0001 Erythema cure: 31.1% (lotilaner) vs. 9.0% (vehicle), *p* < 0.0001	No related SAEs; most commonly reported drug-related AE: instillation site pain: 7.9% (lotilaner) vs. 6.7% (vehicle)

AE: adverse event; SAE: serious adverse event; TEAE: treatment-emergent adverse event; OU: both eyes; BID: twice daily.

**Table 2:** Grading scales used in pivotal Saturn-1 and Saturn-2 clinical trials.

	Grade 0	Grade 1	Grade 2	Grade 3	Grade 4
Collarettes	0–2 lashes per eyelid with collarettes	3–10 lashes per eyelid with collarettes	>10 but <1/3 of the lashes per eyelid with collarettes	≥1/3 but <2/3 of the lashes per eyelid with collarettes	≥2/3 of the lashes per eyelid with collarettes
Erythema	None Normal age-related lid coloration	Mild Pink capillary involvement along the lid edge, no patches of confluent capillary redness throughout the lid edge	Moderate Deep pink or red confluent capillary redness present locally along the lid edge	Severe Deep red, diffuse confluent capillary redness present along the lid edge	N/A

## Data Availability

Not applicable.
